# Chlorido(chloro­difluoro­acetato-κ*O*)bis(1,10-phenanthroline-κ^2^ *N*,*N*′)manganese(II)

**DOI:** 10.1107/S1600536808010829

**Published:** 2008-04-23

**Authors:** Kong Mun Lo, Seik Weng Ng

**Affiliations:** a Department of Chemistry, University of Malaya, 50603 Kuala Lumpur, Malaysia

## Abstract

The chloride and chloro­difluoro­acetate anions occupy *cis* positions in the octa­hedral coordination geometry of the title compound, [Mn(C_2_ClF_2_O_2_)Cl(C_12_H_8_N_2_)_2_]. The two *N*-heterocycles both chelate the metal atom.

## Related literature

For isostructural chlorido(1,10-phenanthroline)(trichloro­acetato)manganese(II), see: Chen *et al.* (2006 ▶).
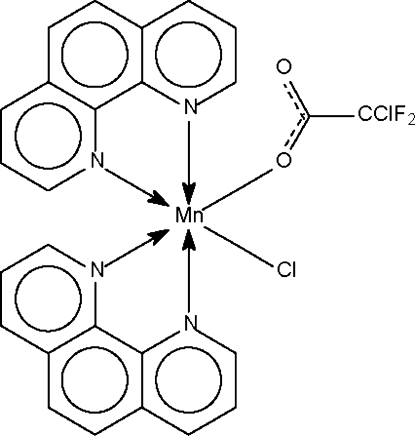


## Experimental

### 

#### Crystal data

[Mn(C_2_ClF_2_O_2_)Cl(C_12_H_8_N_2_)_2_]
*M* *_r_* = 580.27
Monoclinic, 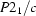

*a* = 16.8822 (4) Å
*b* = 10.3781 (3) Å
*c* = 14.8364 (5) Å
β = 108.813 (2)°
*V* = 2460.5 (1) Å^3^
*Z* = 4
Mo *K*α radiation
μ = 0.80 mm^−1^
*T* = 100 (2) K
0.20 × 0.15 × 0.10 mm

#### Data collection

Bruker SMART APEXII diffractometer
Absorption correction: multi-scan (*SADABS*; Sheldrick, 1996 ▶) *T*_min_ = 0.856, *T*_max_ = 0.924
30077 measured reflections
5628 independent reflections
3863 reflections with *I* > 2σ(*I*)
*R*_int_ = 0.104

#### Refinement

*R*[*F* ^2^ > 2σ(*F* ^2^)] = 0.057
*wR*(*F* ^2^) = 0.165
*S* = 1.04
5628 reflections
334 parameters
H-atom parameters constrained
Δρ_max_ = 1.00 e Å^−3^
Δρ_min_ = −0.63 e Å^−3^

### 

Data collection: *APEX2* (Bruker, 2007 ▶); cell refinement: *SAINT* (Bruker, 2007 ▶); data reduction: *SAINT*; program(s) used to solve structure: *SHELXS97* (Sheldrick, 2008 ▶); program(s) used to refine structure: *SHELXL97* (Sheldrick, 2008 ▶); molecular graphics: *X-SEED* (Barbour, 2001 ▶); software used to prepare material for publication: *publCIF* (Westrip, 2008 ▶).

## Supplementary Material

Crystal structure: contains datablocks I, global. DOI: 10.1107/S1600536808010829/sg2236sup1.cif

Structure factors: contains datablocks I. DOI: 10.1107/S1600536808010829/sg2236Isup2.hkl

Additional supplementary materials:  crystallographic information; 3D view; checkCIF report

## Figures and Tables

**Table:** 

Mn1—O1	2.143 (2)
Mn1—N1	2.283 (3)
Mn1—N2	2.277 (3)
Mn1—N3	2.305 (3)
Mn1—N4	2.295 (3)
Mn1—Cl1	2.443 (1)

**Table:** 

O1—Mn1—N1	110.1 (1)
O1—Mn1—N2	88.1 (1)
O1—Mn1—N3	82.9 (1)
O1—Mn1—N4	153.8 (1)
O1—Mn1—Cl1	101.1 (1)
N1—Mn1—N2	72.6 (1)
N1—Mn1—N3	159.7 (1)
N1—Mn1—N4	92.1 (1)
N1—Mn1—Cl1	90.4 (1)
N2—Mn1—N3	93.0 (1)
N2—Mn1—N4	85.2 (1)
N2—Mn1—Cl1	162.7 (1)
N3—Mn1—N4	72.2 (1)
N3—Mn1—Cl1	102.6 (1)
N4—Mn1—Cl1	92.4 (1)

**Fig. 1.**
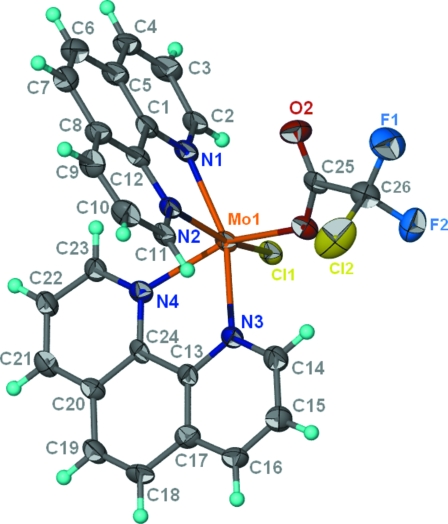
70% Probability thermal ellipsoid plot (Barbour, 2001) of Mn(C12H8N2)2(C2ClF2O2)Cl. Hydrogen atoms are drawn as spheres of arbitrary radii.

## supplementary crystallographic information

### Comment

Manganese dichloride typically reacts with carboxylate anions in the presence of a neutral α,α-dimine ligand (such as 1,10-phenanthroline) to furnish the expected manganese dicarboxylate as the 1:2 adduct of the *N*-heterocycle. In the case of the reaction with the trichloroacetate anion, only one chloride is displaced. Chlorido-bis(1,10-phenanthroline)(trichloroacetato)manganese exists as a monomeric compound; the crystal structure displays π–π interactions that appear to stabilize the structure (Chen *et al.*, 2006). Replacing the trichloroacetate anion by the chlorodifluoroacetate anion furnishes an isostructural compound (Scheme I, Fig. 1).

### Experimental

Manganese dichloride dihydrate (0.5 g, 3 mmol) was dissolved in ethanol and chlorodifluoroacetic acid (0.3 ml, 3 mol) was added. The mixture was heated briefly, after which 1,10-phenanthroline (1.6 g, 6 mmol) was added. The solution when allowed to cool yielded yellow crystals.

### Refinement

Carbon-bound H-atoms were placed in calculated positions (C—H 0.95 Å) and were included in the refinement in the riding model approximation, with *U*(H) set to 1.2*U*_eq_(C).

### Crystal data

**Table:**

[Mn(C_2_ClF_2_O_2_)Cl(C_12_H_8_N_2_)_2_]	*F*_000_ = 1172
*M**_r_* = 580.27	*D*_x_ = 1.566 Mg m^−^^3^
Monoclinic, *P*2_1_/*c*	Mo *K*α radiation λ = 0.71073 Å
Hall symbol: -P 2ybc	Cell parameters from 2908 reflections
*a* = 16.8822 (4) Å	θ = 2.3–21.4º
*b* = 10.3781 (3) Å	µ = 0.80 mm^−^^1^
*c* = 14.8364 (5) Å	*T* = 100 (2) K
β = 108.813 (2)º	Irregular block, yellow
*V* = 2460.5 (1) Å^3^	0.20 × 0.15 × 0.10 mm
*Z* = 4	

### Data collection

**Table:**

Bruker SMART APEX diffractometer	5628 independent reflections
Radiation source: fine-focus sealed tube	3863 reflections with *I* > 2σ(*I*)
Monochromator: graphite	*R*_int_ = 0.104
*T* = 100(2) K	θ_max_ = 27.5º
ω scans	θ_min_ = 1.3º
Absorption correction: multi-scan(SADABS; Sheldrick, 1996)	*h* = −21→21
*T*_min_ = 0.856, *T*_max_ = 0.924	*k* = −13→13
30077 measured reflections	*l* = −19→14

### Refinement

**Table:**

Refinement on *F*^2^	Secondary atom site location: difference Fourier map
Least-squares matrix: full	Hydrogen site location: inferred from neighbouring sites
*R*[*F*^2^ > 2σ(*F*^2^)] = 0.057	H-atom parameters constrained
*wR*(*F*^2^) = 0.165	*w* = 1/[σ^2^(*F*_o_^2^) + (0.0868*P*)^2^] where *P* = (*F*_o_^2^ + 2*F*_c_^2^)/3
*S* = 1.04	(Δ/σ)_max_ = 0.001
5628 reflections	Δρ_max_ = 1.00 e Å^−^^3^
334 parameters	Δρ_min_ = −0.62 e Å^−^^3^
Primary atom site location: structure-invariant direct methods	Extinction correction: none

### Fractional atomic coordinates and isotropic or equivalent isotropic displacement parameters (Å^2^)

**Table:**

	*x*	*y*	*z*	*U*_iso_*/*U*_eq_	
Mn1	0.28449 (3)	0.63507 (5)	0.17308 (4)	0.02130 (16)	
Cl1	0.33600 (5)	0.60920 (8)	0.03799 (6)	0.0247 (2)	
Cl2	0.21097 (8)	1.04489 (11)	0.29372 (9)	0.0536 (3)	
F1	0.09738 (14)	1.0724 (2)	0.1347 (2)	0.0545 (8)	
F2	0.22337 (14)	1.0904 (2)	0.13132 (18)	0.0402 (6)	
O1	0.24710 (14)	0.8334 (2)	0.15829 (18)	0.0264 (6)	
O2	0.10701 (14)	0.8221 (2)	0.1178 (2)	0.0330 (6)	
N1	0.17254 (16)	0.5061 (3)	0.1004 (2)	0.0240 (6)	
N2	0.21023 (16)	0.6109 (3)	0.2757 (2)	0.0230 (6)	
N3	0.39971 (15)	0.7160 (3)	0.2897 (2)	0.0219 (6)	
N4	0.36197 (16)	0.4637 (3)	0.2525 (2)	0.0215 (6)	
C1	0.1249 (2)	0.4714 (3)	0.1547 (3)	0.0239 (7)	
C2	0.1524 (2)	0.4572 (3)	0.0131 (3)	0.0275 (8)	
H2	0.1857	0.4800	−0.0254	0.033*	
C3	0.0848 (2)	0.3739 (3)	−0.0250 (3)	0.0338 (9)	
H3	0.0720	0.3432	−0.0885	0.041*	
C4	0.0376 (2)	0.3369 (3)	0.0295 (3)	0.0312 (9)	
H4	−0.0080	0.2793	0.0049	0.037*	
C5	0.0572 (2)	0.3857 (3)	0.1237 (3)	0.0279 (8)	
C6	0.0103 (2)	0.3542 (3)	0.1854 (3)	0.0328 (9)	
H6	−0.0356	0.2963	0.1638	0.039*	
C7	0.0297 (2)	0.4050 (4)	0.2738 (3)	0.0313 (9)	
H7	−0.0018	0.3813	0.3140	0.038*	
C8	0.0975 (2)	0.4944 (3)	0.3072 (3)	0.0266 (8)	
C9	0.1187 (2)	0.5535 (3)	0.3974 (3)	0.0294 (8)	
H9	0.0885	0.5332	0.4397	0.035*	
C10	0.1829 (2)	0.6399 (3)	0.4236 (3)	0.0316 (8)	
H10	0.1973	0.6819	0.4836	0.038*	
C11	0.2276 (2)	0.6658 (3)	0.3604 (3)	0.0248 (7)	
H11	0.2724	0.7257	0.3795	0.030*	
C12	0.14495 (19)	0.5264 (3)	0.2484 (3)	0.0228 (7)	
C13	0.45711 (19)	0.6274 (3)	0.3381 (2)	0.0217 (7)	
C14	0.4167 (2)	0.8390 (3)	0.3096 (3)	0.0274 (8)	
H14	0.3766	0.9014	0.2767	0.033*	
C15	0.4910 (2)	0.8821 (3)	0.3767 (3)	0.0299 (8)	
H15	0.5006	0.9716	0.3891	0.036*	
C16	0.5496 (2)	0.7935 (3)	0.4243 (3)	0.0282 (8)	
H16	0.6007	0.8210	0.4696	0.034*	
C17	0.5338 (2)	0.6622 (3)	0.4060 (3)	0.0251 (8)	
C18	0.5921 (2)	0.5629 (3)	0.4517 (3)	0.0291 (8)	
H18	0.6446	0.5861	0.4962	0.035*	
C19	0.5738 (2)	0.4374 (3)	0.4329 (3)	0.0280 (8)	
H19	0.6135	0.3737	0.4643	0.034*	
C20	0.4951 (2)	0.3985 (3)	0.3662 (3)	0.0241 (7)	
C21	0.4724 (2)	0.2692 (3)	0.3466 (3)	0.0271 (8)	
H21	0.5097	0.2025	0.3778	0.033*	
C22	0.3961 (2)	0.2393 (3)	0.2822 (3)	0.0284 (8)	
H22	0.3795	0.1520	0.2689	0.034*	
C23	0.3430 (2)	0.3395 (3)	0.2362 (3)	0.0249 (8)	
H23	0.2906	0.3178	0.1909	0.030*	
C24	0.43732 (19)	0.4937 (3)	0.3181 (2)	0.0211 (7)	
C25	0.1748 (2)	0.8752 (3)	0.1456 (2)	0.0234 (7)	
C26	0.1740 (2)	1.0211 (3)	0.1694 (3)	0.0330 (9)	

### Atomic displacement parameters (Å^2^)

**Table:**

	*U*^11^	*U*^22^	*U*^33^	*U*^12^	*U*^13^	*U*^23^
Mn1	0.0192 (3)	0.0265 (3)	0.0161 (3)	−0.00035 (19)	0.0028 (2)	−0.0001 (2)
Cl1	0.0231 (4)	0.0310 (4)	0.0189 (5)	0.0005 (3)	0.0053 (3)	0.0006 (3)
Cl2	0.0682 (7)	0.0614 (7)	0.0398 (7)	−0.0225 (6)	0.0291 (6)	−0.0250 (5)
F1	0.0351 (12)	0.0376 (13)	0.086 (2)	0.0129 (10)	0.0131 (13)	0.0026 (13)
F2	0.0446 (13)	0.0359 (12)	0.0427 (16)	−0.0083 (10)	0.0178 (11)	0.0021 (11)
O1	0.0224 (12)	0.0323 (13)	0.0221 (14)	0.0033 (9)	0.0038 (10)	0.0028 (10)
O2	0.0236 (12)	0.0375 (14)	0.0354 (17)	−0.0043 (10)	0.0061 (12)	−0.0090 (12)
N1	0.0213 (13)	0.0294 (15)	0.0185 (16)	0.0014 (11)	0.0024 (12)	−0.0009 (12)
N2	0.0199 (13)	0.0282 (15)	0.0185 (16)	0.0015 (11)	0.0028 (12)	0.0013 (12)
N3	0.0200 (13)	0.0265 (15)	0.0174 (16)	−0.0022 (11)	0.0036 (12)	0.0019 (11)
N4	0.0207 (13)	0.0280 (15)	0.0158 (16)	−0.0024 (11)	0.0057 (12)	−0.0013 (11)
C1	0.0208 (15)	0.0279 (17)	0.0204 (19)	0.0042 (13)	0.0032 (14)	0.0028 (14)
C2	0.0279 (17)	0.0340 (19)	0.017 (2)	−0.0003 (14)	0.0022 (15)	−0.0027 (15)
C3	0.034 (2)	0.037 (2)	0.024 (2)	−0.0029 (16)	0.0000 (17)	−0.0069 (16)
C4	0.0240 (17)	0.0299 (19)	0.032 (2)	−0.0014 (14)	−0.0015 (16)	−0.0027 (16)
C5	0.0242 (17)	0.0295 (18)	0.025 (2)	0.0015 (14)	0.0017 (15)	−0.0007 (15)
C6	0.0222 (17)	0.035 (2)	0.037 (2)	−0.0039 (14)	0.0042 (17)	0.0015 (17)
C7	0.0267 (18)	0.037 (2)	0.033 (2)	0.0002 (15)	0.0130 (17)	0.0044 (17)
C8	0.0221 (16)	0.0308 (18)	0.025 (2)	0.0011 (14)	0.0044 (15)	0.0028 (15)
C9	0.0291 (18)	0.040 (2)	0.020 (2)	0.0007 (15)	0.0101 (16)	0.0041 (16)
C10	0.0336 (19)	0.040 (2)	0.020 (2)	0.0023 (16)	0.0070 (16)	−0.0016 (16)
C11	0.0225 (16)	0.0343 (18)	0.0140 (18)	−0.0009 (13)	0.0009 (14)	−0.0014 (14)
C12	0.0177 (15)	0.0279 (17)	0.0197 (19)	0.0032 (13)	0.0018 (14)	0.0015 (14)
C13	0.0211 (15)	0.0271 (17)	0.0172 (18)	−0.0006 (13)	0.0067 (14)	0.0018 (14)
C14	0.0259 (17)	0.0265 (18)	0.028 (2)	−0.0006 (13)	0.0063 (16)	0.0005 (15)
C15	0.0263 (17)	0.0292 (18)	0.030 (2)	−0.0073 (14)	0.0042 (16)	−0.0041 (16)
C16	0.0240 (16)	0.0343 (19)	0.024 (2)	−0.0074 (14)	0.0048 (15)	−0.0008 (15)
C17	0.0229 (16)	0.0341 (19)	0.0187 (19)	−0.0043 (14)	0.0071 (15)	0.0009 (15)
C18	0.0202 (16)	0.037 (2)	0.025 (2)	−0.0021 (14)	0.0003 (15)	−0.0001 (16)
C19	0.0235 (16)	0.0338 (19)	0.022 (2)	0.0043 (14)	0.0014 (15)	0.0031 (15)
C20	0.0209 (16)	0.0340 (18)	0.0159 (18)	0.0020 (14)	0.0042 (14)	0.0017 (14)
C21	0.0263 (17)	0.0274 (18)	0.026 (2)	0.0048 (14)	0.0061 (15)	0.0028 (15)
C22	0.0268 (17)	0.0271 (18)	0.028 (2)	0.0018 (14)	0.0047 (15)	0.0000 (15)
C23	0.0213 (16)	0.0295 (18)	0.023 (2)	−0.0012 (13)	0.0055 (15)	0.0006 (14)
C24	0.0206 (15)	0.0303 (17)	0.0134 (17)	0.0002 (13)	0.0070 (13)	0.0025 (13)
C25	0.0224 (16)	0.0364 (19)	0.0107 (17)	0.0001 (14)	0.0045 (14)	0.0008 (14)
C26	0.0266 (17)	0.037 (2)	0.035 (2)	0.0007 (15)	0.0089 (17)	−0.0015 (17)

### Geometric parameters (Å, °)

**Table:**

Mn1—O1	2.143 (2)	C7—C8	1.432 (5)
Mn1—N1	2.283 (3)	C7—H7	0.9500
Mn1—N2	2.277 (3)	C8—C12	1.401 (5)
Mn1—N3	2.305 (3)	C8—C9	1.410 (5)
Mn1—N4	2.295 (3)	C9—C10	1.363 (5)
Mn1—Cl1	2.443 (1)	C9—H9	0.9500
Cl2—C26	1.764 (4)	C10—C11	1.405 (5)
F1—C26	1.339 (4)	C10—H10	0.9500
F2—C26	1.355 (4)	C11—H11	0.9500
O1—C25	1.250 (4)	C13—C17	1.407 (5)
O2—C25	1.217 (4)	C13—C24	1.436 (4)
N1—C2	1.330 (5)	C14—C15	1.400 (5)
N1—C1	1.358 (5)	C14—H14	0.9500
N2—C11	1.324 (5)	C15—C16	1.368 (5)
N2—C12	1.363 (4)	C15—H15	0.9500
N3—C14	1.321 (4)	C16—C17	1.398 (5)
N3—C13	1.361 (4)	C16—H16	0.9500
N4—C23	1.331 (4)	C17—C18	1.436 (5)
N4—C24	1.365 (4)	C18—C19	1.346 (5)
C1—C5	1.403 (5)	C18—H18	0.9500
C1—C12	1.440 (5)	C19—C20	1.435 (5)
C2—C3	1.398 (5)	C19—H19	0.9500
C2—H2	0.9500	C20—C21	1.399 (5)
C3—C4	1.362 (6)	C20—C24	1.410 (4)
C3—H3	0.9500	C21—C22	1.369 (5)
C4—C5	1.422 (5)	C21—H21	0.9500
C4—H4	0.9500	C22—C23	1.398 (5)
C5—C6	1.428 (6)	C22—H22	0.9500
C6—C7	1.353 (6)	C23—H23	0.9500
C6—H6	0.9500	C25—C26	1.556 (5)
			
O1—Mn1—N1	110.1 (1)	C9—C10—C11	119.0 (4)
O1—Mn1—N2	88.1 (1)	C9—C10—H10	120.5
O1—Mn1—N3	82.9 (1)	C11—C10—H10	120.5
O1—Mn1—N4	153.8 (1)	N2—C11—C10	123.3 (3)
O1—Mn1—Cl1	101.1 (1)	N2—C11—H11	118.4
N1—Mn1—N2	72.6 (1)	C10—C11—H11	118.3
N1—Mn1—N3	159.7 (1)	N2—C12—C8	122.5 (3)
N1—Mn1—N4	92.1 (1)	N2—C12—C1	117.2 (3)
N1—Mn1—Cl1	90.4 (1)	C8—C12—C1	120.2 (3)
N2—Mn1—N3	93.0 (1)	N3—C13—C17	122.6 (3)
N2—Mn1—N4	85.2 (1)	N3—C13—C24	117.8 (3)
N2—Mn1—Cl1	162.7 (1)	C17—C13—C24	119.7 (3)
N3—Mn1—N4	72.2 (1)	N3—C14—C15	123.2 (3)
N3—Mn1—Cl1	102.6 (1)	N3—C14—H14	118.4
N4—Mn1—Cl1	92.4 (1)	C15—C14—H14	118.4
C25—O1—Mn1	126.1 (2)	C16—C15—C14	119.0 (3)
C2—N1—C1	117.6 (3)	C16—C15—H15	120.5
C2—N1—Mn1	126.6 (2)	C14—C15—H15	120.5
C1—N1—Mn1	115.7 (2)	C15—C16—C17	119.6 (3)
C11—N2—C12	117.9 (3)	C15—C16—H16	120.2
C11—N2—Mn1	125.9 (2)	C17—C16—H16	120.2
C12—N2—Mn1	116.0 (2)	C16—C17—C13	117.6 (3)
C14—N3—C13	117.9 (3)	C16—C17—C18	123.3 (3)
C14—N3—Mn1	126.2 (2)	C13—C17—C18	119.1 (3)
C13—N3—Mn1	115.8 (2)	C19—C18—C17	121.3 (3)
C23—N4—C24	117.7 (3)	C19—C18—H18	119.3
C23—N4—Mn1	126.4 (2)	C17—C18—H18	119.3
C24—N4—Mn1	115.8 (2)	C18—C19—C20	121.0 (3)
N1—C1—C5	123.3 (3)	C18—C19—H19	119.5
N1—C1—C12	117.9 (3)	C20—C19—H19	119.5
C5—C1—C12	118.7 (3)	C21—C20—C24	117.9 (3)
N1—C2—C3	123.3 (4)	C21—C20—C19	123.0 (3)
N1—C2—H2	118.4	C24—C20—C19	119.1 (3)
C3—C2—H2	118.4	C22—C21—C20	119.7 (3)
C4—C3—C2	119.5 (4)	C22—C21—H21	120.2
C4—C3—H3	120.3	C20—C21—H21	120.2
C2—C3—H3	120.3	C21—C22—C23	118.9 (3)
C3—C4—C5	119.2 (3)	C21—C22—H22	120.6
C3—C4—H4	120.4	C23—C22—H22	120.6
C5—C4—H4	120.4	N4—C23—C22	123.5 (3)
C1—C5—C4	117.0 (3)	N4—C23—H23	118.2
C1—C5—C6	119.8 (4)	C22—C23—H23	118.2
C4—C5—C6	123.1 (3)	N4—C24—C20	122.3 (3)
C7—C6—C5	121.4 (3)	N4—C24—C13	117.9 (3)
C7—C6—H6	119.3	C20—C24—C13	119.8 (3)
C5—C6—H6	119.3	O2—C25—O1	131.3 (3)
C6—C7—C8	120.3 (4)	O2—C25—C26	116.1 (3)
C6—C7—H7	119.9	O1—C25—C26	112.5 (3)
C8—C7—H7	119.9	F1—C26—F2	106.1 (3)
C12—C8—C9	117.8 (3)	F1—C26—C25	112.2 (3)
C12—C8—C7	119.5 (3)	F2—C26—C25	111.7 (3)
C9—C8—C7	122.7 (3)	F1—C26—Cl2	108.6 (3)
C10—C9—C8	119.5 (4)	F2—C26—Cl2	107.7 (2)
C10—C9—H9	120.3	C25—C26—Cl2	110.3 (3)
C8—C9—H9	120.3		
			
N2—Mn1—O1—C25	−45.9 (3)	C12—C8—C9—C10	1.0 (5)
N1—Mn1—O1—C25	24.8 (3)	C7—C8—C9—C10	−178.5 (3)
N4—Mn1—O1—C25	−121.0 (3)	C8—C9—C10—C11	−1.4 (5)
N3—Mn1—O1—C25	−139.1 (3)	C12—N2—C11—C10	0.9 (5)
Cl1—Mn1—O1—C25	119.4 (3)	Mn1—N2—C11—C10	−175.2 (2)
O1—Mn1—N1—C2	96.7 (3)	C9—C10—C11—N2	0.4 (5)
N2—Mn1—N1—C2	178.0 (3)	C11—N2—C12—C8	−1.3 (5)
N4—Mn1—N1—C2	−97.7 (3)	Mn1—N2—C12—C8	175.2 (2)
N3—Mn1—N1—C2	−135.8 (3)	C11—N2—C12—C1	177.6 (3)
Cl1—Mn1—N1—C2	−5.3 (3)	Mn1—N2—C12—C1	−5.9 (4)
O1—Mn1—N1—C1	−87.0 (2)	C9—C8—C12—N2	0.4 (5)
N2—Mn1—N1—C1	−5.7 (2)	C7—C8—C12—N2	179.9 (3)
N4—Mn1—N1—C1	78.7 (2)	C9—C8—C12—C1	−178.5 (3)
N3—Mn1—N1—C1	40.5 (4)	C7—C8—C12—C1	1.0 (5)
Cl1—Mn1—N1—C1	171.1 (2)	N1—C1—C12—N2	0.7 (4)
O1—Mn1—N2—C11	−66.0 (3)	C5—C1—C12—N2	−179.0 (3)
N1—Mn1—N2—C11	−177.7 (3)	N1—C1—C12—C8	179.7 (3)
N4—Mn1—N2—C11	88.6 (3)	C5—C1—C12—C8	0.0 (5)
N3—Mn1—N2—C11	16.7 (3)	C14—N3—C13—C17	−1.5 (5)
Cl1—Mn1—N2—C11	171.2 (2)	Mn1—N3—C13—C17	174.8 (3)
O1—Mn1—N2—C12	117.8 (2)	C14—N3—C13—C24	178.3 (3)
N1—Mn1—N2—C12	6.1 (2)	Mn1—N3—C13—C24	−5.3 (4)
N4—Mn1—N2—C12	−87.6 (2)	C13—N3—C14—C15	0.8 (5)
N3—Mn1—N2—C12	−159.4 (2)	Mn1—N3—C14—C15	−175.1 (3)
Cl1—Mn1—N2—C12	−5.0 (4)	N3—C14—C15—C16	0.3 (6)
O1—Mn1—N3—C14	−6.1 (3)	C14—C15—C16—C17	−0.8 (6)
N2—Mn1—N3—C14	−93.8 (3)	C15—C16—C17—C13	0.1 (5)
N1—Mn1—N3—C14	−137.4 (3)	C15—C16—C17—C18	179.2 (4)
N4—Mn1—N3—C14	−177.8 (3)	N3—C13—C17—C16	1.1 (5)
Cl1—Mn1—N3—C14	93.7 (3)	C24—C13—C17—C16	−178.8 (3)
O1—Mn1—N3—C13	177.9 (2)	N3—C13—C17—C18	−178.1 (3)
N2—Mn1—N3—C13	90.2 (2)	C24—C13—C17—C18	2.1 (5)
N1—Mn1—N3—C13	46.6 (4)	C16—C17—C18—C19	179.0 (4)
N4—Mn1—N3—C13	6.1 (2)	C13—C17—C18—C19	−1.9 (6)
Cl1—Mn1—N3—C13	−82.3 (2)	C17—C18—C19—C20	0.0 (6)
O1—Mn1—N4—C23	159.6 (3)	C18—C19—C20—C21	−178.0 (4)
N2—Mn1—N4—C23	83.7 (3)	C18—C19—C20—C24	1.7 (5)
N1—Mn1—N4—C23	11.4 (3)	C24—C20—C21—C22	0.2 (5)
N3—Mn1—N4—C23	178.4 (3)	C19—C20—C21—C22	179.9 (4)
Cl1—Mn1—N4—C23	−79.1 (3)	C20—C21—C22—C23	1.0 (6)
O1—Mn1—N4—C24	−25.2 (4)	C24—N4—C23—C22	−0.3 (5)
N2—Mn1—N4—C24	−101.0 (2)	Mn1—N4—C23—C22	174.8 (3)
N1—Mn1—N4—C24	−173.4 (2)	C21—C22—C23—N4	−1.0 (6)
N3—Mn1—N4—C24	−6.4 (2)	C23—N4—C24—C20	1.6 (5)
Cl1—Mn1—N4—C24	96.1 (2)	Mn1—N4—C24—C20	−174.1 (3)
C2—N1—C1—C5	1.2 (5)	C23—N4—C24—C13	−178.2 (3)
Mn1—N1—C1—C5	−175.5 (2)	Mn1—N4—C24—C13	6.1 (4)
C2—N1—C1—C12	−178.5 (3)	C21—C20—C24—N4	−1.5 (5)
Mn1—N1—C1—C12	4.8 (4)	C19—C20—C24—N4	178.8 (3)
C1—N1—C2—C3	0.7 (5)	C21—C20—C24—C13	178.3 (3)
Mn1—N1—C2—C3	176.9 (3)	C19—C20—C24—C13	−1.4 (5)
N1—C2—C3—C4	−1.8 (5)	N3—C13—C24—N4	−0.5 (5)
C2—C3—C4—C5	1.0 (5)	C17—C13—C24—N4	179.3 (3)
N1—C1—C5—C4	−1.8 (5)	N3—C13—C24—C20	179.7 (3)
C12—C1—C5—C4	177.9 (3)	C17—C13—C24—C20	−0.5 (5)
N1—C1—C5—C6	180.0 (3)	Mn1—O1—C25—O2	−18.7 (6)
C12—C1—C5—C6	−0.3 (5)	Mn1—O1—C25—C26	161.9 (2)
C3—C4—C5—C1	0.7 (5)	O2—C25—C26—F1	−14.5 (5)
C3—C4—C5—C6	178.8 (3)	O1—C25—C26—F1	164.9 (3)
C1—C5—C6—C7	−0.2 (5)	O2—C25—C26—F2	−133.6 (3)
C4—C5—C6—C7	−178.3 (3)	O1—C25—C26—F2	45.9 (4)
C5—C6—C7—C8	1.2 (5)	O2—C25—C26—Cl2	106.7 (3)
C6—C7—C8—C12	−1.6 (5)	O1—C25—C26—Cl2	−73.8 (3)
C6—C7—C8—C9	177.9 (3)		
